# Thermographic Screening of Beef Cattle Metatarsal Growth Plate Lesions

**DOI:** 10.3390/ani12020191

**Published:** 2022-01-13

**Authors:** Giorgia Fabbri, Matteo Gianesella, Rossella Tessari, Andrea Bassini, Massimo Morgante, Barbara Contiero, Vanessa Faillace, Enrico Fiore

**Affiliations:** 1 Department of Animal Medicine, Productions and Health (MAPS), University of Padua, Viale dell’Università 16, 35020 Legnaro, PD, Italy; giorgia.fabbri@unipd.it (G.F.); rossella.tessari@unipd.it (R.T.); massimo.morgante@unipd.it (M.M.); barbara.contiero@unipd.it (B.C.); vanessa.faillace@unipd.it (V.F.); 2 MSD Animal Health Srl, 20090 Segrate, MI, Italy; andrea.bassini@merck.com

**Keywords:** beef cattle, growth plate lesions, infrared thermography, physitis

## Abstract

**Simple Summary:**

Young beef bulls are predisposed to develop diseases of the growing skeleton, especially growth plate lesions. These lesions jeopardize both welfare and production, often leading to anticipated culling and diminished weight gain. However, beef cattle are prey animals and do not exhibit signs of pain and lameness until the disease becomes extensive and severe. Fast methods to screen for growth plate lesions presence could therefore lead to prompt treatment of the affected animals, enhancing recovery and diminishing losses. The aim of the present study was to examine the potential of infrared thermography as a non-invasive tool for rapidly screening beef bulls for the presence of growth plate lesions. Here, 20 Charolais and Limousine beef bulls affected by growth plate lesions were screened using infrared thermography. A difference in maximum, mean, and minimum temperatures was detected between healthy and affected growth plate areas, and a difference in mean and maximum temperatures was detected globally in the affected limbs against healthy ones. Infrared thermography could therefore serve as a reliable tool for screening growth plate lesions in beef bulls, permitting rapid detection at pen level and aiding fast and targeted treatment, thus improving animal welfare and production.

**Abstract:**

Lameness represents one of the main causes of decreased productive performance and impaired animal welfare in the bovine industry. Young beef bulls are predisposed to develop diseases of the growing skeleton, especially growth plate lesions. Early diagnosis is indispensable for ensuring correct treatment, fast recovery and reduction losses. However, when dealing with beef cattle, this is not always possible. Fast and reliable diagnostic imaging techniques are necessary to improve dealing with lameness in beef animals. The aim of the present study was to examine the potential of thermographic imaging as a non-invasive tool for rapidly screening beef bulls for the presence of growth plate lesions. Here, 20 Charolais and Limousine beef bulls affected by growth plate lesions in one of the rear limbs were selected. IRT was performed on both hind limbs using a digital infrared camera (ThermaCam T420 Model, Flir Systems, Boston, MA, USA), prior to radiographic imaging and clinical examination. The temperature of healthy and affected limbs was measured in two regions: the area correspondent to the growth plate (AR01) and the whole area of the metatarsus (AR02). Growth plate lesions were found to increase the maximum, mean, and minimum temperatures in AR01; and the mean and maximum temperatures in AR02, therefore, indicating the potential of IRT as a reliable, practical tool for screening growth plate lesions in beef bulls.

## 1. Introduction

Lameness is a major problem affecting cattle, it has a significant impact on health and welfare, and has been proven to cause about 16% of the health problems of feedlot cattle [1]. Lameness also leads to a range of production losses [2], often resulting in earlier culling of animals and decreasing the economic value of carcasses. It reduces the time animals spend feeding and leads to lower carcass weight, conformation class, and fat cover class [2,3,4].

Young beef bulls selected for rapid weight gain are particularly susceptible to developing lesions in the growing skeleton, particularly physeal (growth plate) lesions such as physitis [5,6,7]. Physitis is characterized by inflammation of the growth plates and mostly has a multifactorial origin, such as feed pushing for rapid growth, direct trauma to the ossification centers, infection, nutritional imbalances, and housing on slatted floors [8,9,10,11,12]. A critical aspect when dealing with lameness is early detection and diagnosis. Detecting lameness efficiently allows for early intervention, enhancing recovery chances and minimizing losses. Thus, early diagnosis of physitis cases allows for better farm management decision making processes [13,14].

Physitis can represent a confusing picture with significant lameness and often moderate swelling and pain [12,15,16], and when dealing with growth plate lesions, ruling out other causes for lameness such as arthritis and claw affections is important [3].

Radiography (associated with clinical examination) is generally the most used method to identify and confirm growth plate lesions [6,7,12,17,18]. However, the disadvantages associated with radiographic imaging remain important. Besides the well-known issues related to X-ray emissions, costly equipment can represent a limitation for many freelance professionals. The use of radiography, although highly reliable, is both logistically and economically challenging on a large scale and at pen level when dealing with beef animals.

Among the innovative techniques in diagnostic imaging, infrared thermography (IRT) is experiencing a spike of interest amidst practitioners both in human and veterinary medicine [19]. The perks of IRT are that it is low cost, fast and efficient [20]. Moreover, thermal images can be taken at a distance from the subject, avoiding issues associated with capture and confinement [21]. Infrared thermography (IRT) is a non-invasive, quantitative assessment of temperature, producing a pictorial representation of the surface temperature of an object. The color gradient reflects differences in emitted heat [20,21,22]. Numerous studies in animal sciences have been conducted using thermography as a tool to obtain thermal responses in a rapid and noninvasive manner [23,24]. IRT permits the detection of even small changes in temperature with precision [25], and as heat is a cardinal sign of inflammation, caused by an increase in circulation and tissue metabolism [26], this technique has been used both on humans and animals to detect local inflammation due to disease conditions and injury [19].

The aim of the present study was to examine the potential of IRT as a non-invasive tool for rapidly screening beef bulls for the presence of growth plate metatarsal lesions, assessing the sensitivity of thermographic imaging against other diagnostic methods (radiographic imaging and clinical examination) for the early detection and screening of growth plate lesions.

## 2. Materials and Methods

### 2.1. Experimental Design

Animals were selected among those referred for lameness between 2018 and 2020 at the Veterinary Teaching Hospital (OVUD) of the University of Padua (Viale dell’Università 16; 35020 Legnaro, PD, Italy) and were examined on the respective farm.

All the beef animals with radiographically diagnosed growth plate lesions on one metatarsus were included in the study. A total of 20 Charolais and Limousine male beef bulls, coming from an association of farmers in the same geographic area (Veneto region), and with similar animal husbandry practices, were considered. The animals’ mean age was 13.7 ± 3.5 months and the mean live weight was 494.4 ± 80.8 kg.

All of the farms shared similar housing facilities, with concrete slatted floors, and boxes of 8 ± 1.9 animals each. Animals were housed in groups similar for weight, sex and age. All farms also had infirmary boxes where the animals were relocated after being found lame.

In all farms, the diet was provided once a day as a total mixed ration for ad libitum intake, based on 10% feed refusal. Drinking water was also available ad libitum.

Problems related to nutritional imbalances had never been reported on the farms.

### 2.2. On Field Thermographic and Radiographic Imaging Acquisition, and Clinical Evaluations

**Figure 1:**
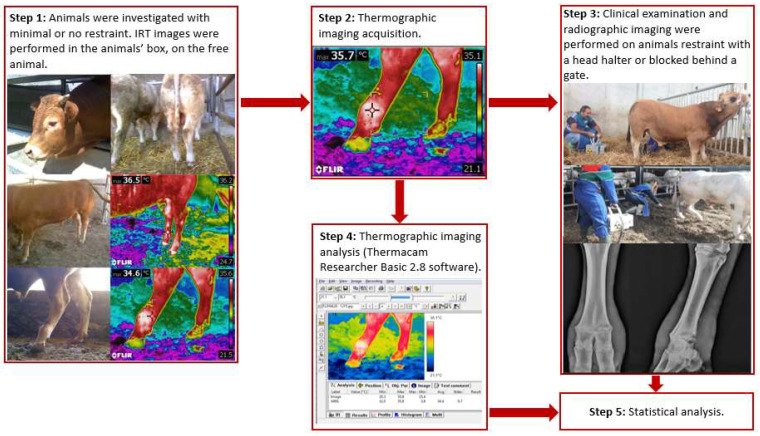
Operational flowchart of the methods in the study.

All of the animals were investigated without sedation. IRT imaging was performed on unbridled animals in the animals’ box or enclosure. A flow chart summarizing the methodological steps of the present study is depicted in Figure 1.

**Figure 2:**
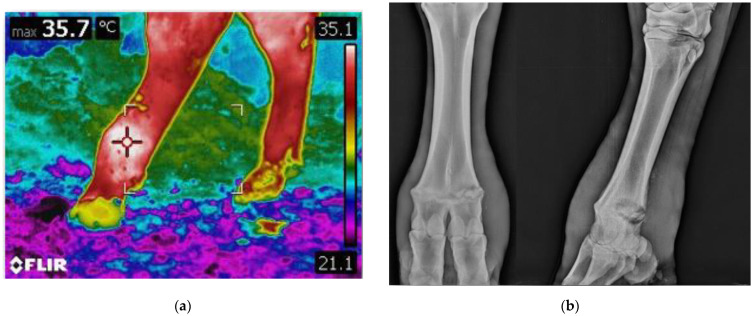
Diagnostic imaging in comparison: (**a**) thermographic imaging of the metatarsal region of the hind limbs, an area of increased brightness is present on the limb on the left, indicating increased skin temperature; (**b**) radiographic imaging of the same limb. Signs of lysis can be appreciated throughout the physis.

Thermographic imaging was performed on both hind limbs for each animal, using a digital infrared camera (ThermaCam T420 Model, Flir Systems, Boston, MA, USA) prior to radiographic imaging and clinical examination. For each animal, multiple IRT images were taken, and for each image, both limbs were included within the same image frame (Figure 2).

All thermographic imaging was performed in a closed, indoor environment, and no cleaning, nor any other intervention, was performed on the limbs prior to diagnostic imaging. The mean ambient temperature was recorded (24.56 ± 5.58 °C). All images were performed at a fixed distance from the subject, to reduce the effects of environmental factors on thermo-graphic readings. The IRT camera had the following settings: temperature range: 10–40 °C; emissivity of skin: 0.98; reflected air temperature (Trifl): 20 °C; distance between the camera and skin surface (Dist): 0.7 m; and view field (FOV): 23°. The detector consisted of a focal plane array (FPA) uncooled microbolometer with the following specifications: 320 × 240 pixels resolution, thermal sensitivity of 0.08 °C (at 30 °C), spatial resolution (IFOV) of 1.3 mrad, spectral range between 7.5, and 13 μm accuracy ±2 °C. Automatic corrections based on user input were conducted for reflected ambient temperature, distance, relative humidity, and atmospheric transmission.

After the thermographic imaging acquisition, the animals underwent a clinical examination and then radiographic imaging. Stance and locomotion score were assessed following guidelines from Shearer et al. [27], and Sprecher et al. [28]. During clinical examination and radiographic imaging, to ensure operators safety and animal welfare the animals were restrained with a rope used as a head halter fastened to the farm’s infrastructures (Figure 1). In the case of particularly aggressive or frightened animals, the animal could also be blocked behind a gate if needed. Clinical examination of the rear limbs was performed, and any skin lesions, swelling, and wounds were recorded. Radiographic examinations of both hindlimbs was performed at the metatarsophalangeal joint level. A diagnostic portable X-ray unit (AJEX140H, AJEX Meditech Ltd., Seoul, Korea) with Carestream CR Pannell Phosphor screens of 24 × 30 cm was used in the study. Images were acquired with a digital portable scanner (DirectView Vita-CR, Carestream Health Inc., Rochester, NY, USA) immediately after being taken. To ensure maximum image quality and operators’ safety without restraining the animals, long handled radiographic panel holders were used during image acquisition. For each radiographed limb, at least two projections were taken: latero-medial (LM) and dorsoplantar (DP) (Figure 2).

All radiographs were performed with the animals in standing position. The exposure parameters used were 6.40–7.20 mAs e 60 kVp and the X-ray generator was kept 1 m from the cassette. Limbs were classified based on their radiographic findings: limbs with no abnormal radiographic signs were considered healthy, while limbs with radiographic signs of lysis or irregularities in the epiphyseal cartilage were considered affected.

### 2.3. Thermographic Imaging Analysis

**Figure 3:**
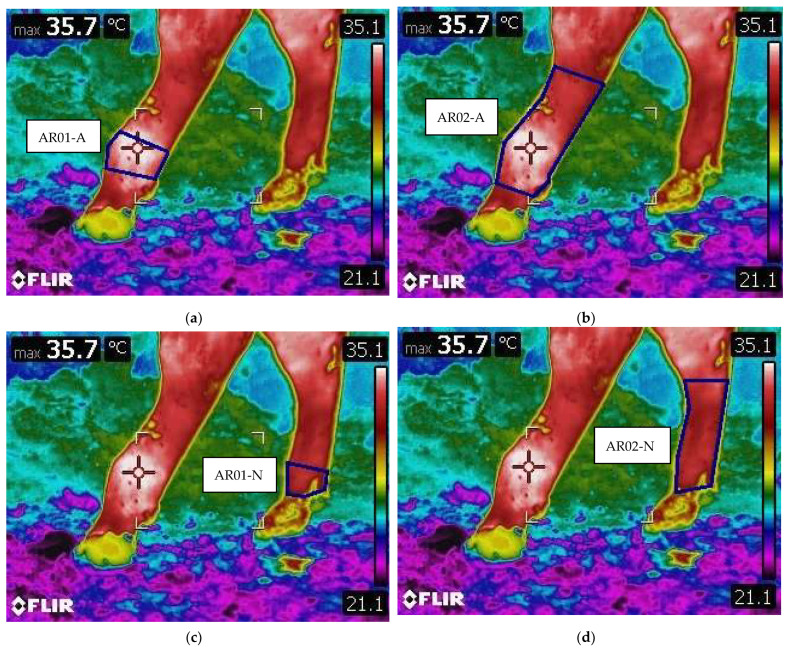
Example of ROI areas, identified by the blue square. (**a**) AR01-A: Growth plate area on the affected metatarsus. (**b**) AR02-A: Whole area of the affected metatarsus. (**c**) AR01-N: Growth plate area on the healthy metatarsus. (**d**) AR02-N: Whole area of the healthy metatarsus.

All of the animals that had similar growth plate lesions, characterized by separation through the physis and only one of the limbs affected while the contralateral was healthy, were included in the study. Thermographic images of the selected animals were evaluated blindly. The temperature of the limbs was measured in four specific regions of interest (ROI). The growth plate area of the affected metatarsus (AR01-A) and of the normal, healthy one (AR02-N), were identified as the area corresponding to the metatarsal distal head, and the whole area of the affected metatarsus (AR02-A) and of the healthy one (AR02-N), were identified as the area included between the dew claws and the fetlock joint area distally, and by the tarsometatarsal joint in the hock area proximally. ROI’s are shown in Figure 3.

For each ROI, thermography software (Thermacam Researcher Basic 2.8 software, FLIR, Wilsonville, OR, USA) was used to evaluate the following parameters: minimum temperature (°C) of the selected area (Tmin), maximum temperature (°C) of the selected area (Tmax), mean temperature (°C) of the selected area (Tmean), difference between Tmax-Tmin (ΔT), and standard deviation (SD).

### 2.4. Statistical Analysis

The statistical analysis was carried out using software package SAS 9.4 (SAS Inst. Inc., Cary, NC, USA). All of the data were tested for normality of distribution using the Kolmogorov−Smirnov test. As for each animal, multiple IRT images were present, a linear mixed model was performed to be able to take into account the individual factor, and the animal effect was included in the mixed model as repeated and random effect. Healthy versus affected limbs’ ROI’s were tested for the temperature parameters (Tmin, Tmax, Tmean and ΔT). Specifically, AR01-N temperature parameters were tested against AR01-A ones, and AR02-N parameters were tested against AR02-A. 

## 3. Results

Growth plate lesions of the animals included in the study were distributed as follows: 10 left limbs and 10 right ones. 

The locomotion score median ± IQR was found to be 4 ± 1.

The parameters, mean, and standard deviation of IRT images are summarized in Table 1.

Differences were found for healthy limbs against diseased ones in both AR01 and AR02. Statistically significant differences were found for maximum and mean, (*p* < 0.0001), and for minimum (*p* < 0.005) temperatures in AR01 between healthy limbs and limbs with growth plate defects. In AR02, statistically significant differences were present in the mean (*p* < 0.0001) and maximum (*p* < 0.005) temperatures among healthy against diseased limbs. In addition, the maximum, mean and minimum temperature differed between AR01-A and AR01-N of >1.8 (with T-max reaching a difference of 2); while in AR02, the mean and minimum temperature differed between AR02-A and AR02-N of >1.8. The results are summarized in Table 2.

## 4. Discussion

The present study represents a pilot investigation in screening for beef bull’s growth plate lesions using IRT diagnostic imaging. IRT images from 20 animals radiographically diagnosed with a metatarsal growth plate lesion on one of their hindlimbs were analyzed for temperature differences between the affected and healthy limbs. 

IRT was proven useful in detecting changes in blood flow and localized inflammatory conditions [19,29]. Growth plate lesions, such as physitis, are a common issue of young beef bulls, especially those selected for rapid weight gain [5,6,7]. Physitis is characterized by inflammation of the growth plate, and it affects mainly the immature long bones of both fore and hindlimbs [8,16]. As the intensity of infrared radiation emitted by the skin was proven to be directly proportional to the metabolic processes occurring in related surfaces and is associated with a simultaneous increase in blood supply to a given area [30], the inflammatory condition that characterizes growth plate areas during physitis would result in an increase in blood flow, and therefore emitted heat. The presence of growth plate lesions was found to be associated with a significant increase in skin temperatures both for AR01-N against AR01-A, and for AR02-N against AR02-A. Significative difference were found for maximum, mean and minimum temperatures in AR01, and for mean and maximum temperatures in AR02. Moreover, the affected limbs showed a higher mean when compared to the healthy ones for the parameters with significative *p*-values.

When assessing temperature differences between areas, the most reliable factors in the present study were the maximum and mean temperatures. These parameters showed differences in both AR01 and AR02 areas. When examining the AR02 area, the mean temperature showed significant *p*-values (*p* < 0.0001) when used to screen for growth plate lesions. Maximum temperature, although having a lower *p*-value than mean temperature, was significant (*p* < 0.005) too. Minimum temperature, however, was found to be significant only when examining the lesion area (AR01) specifically, and not the whole metatarsus area (AR02). 

The inflammatory process that characterizes physitis is mainly localized to the area corresponding to the growth plate, thus AR01. It is possible that when the limb is screened broadly (area AR02), echoes of the inflammation can determine an elevation in maximum and mean temperatures. The infrared radiation emitted by the lesion area will be higher than the healthy one because of the inflammatory process, and will therefore be registered by IRT, thus influencing maximum and mean temperatures. However, the minimum temperature will be the similar to the one emitted by the healthy limb, because the baseline is not affected. It is therefore observable how minimum temperature is a parameter that is less subjected to variation caused by an inflammatory status than the other two. Moreover, when dealing with minimum temperatures, there could be other confounding factors present on the limb that are not presently being taken into account, such as the presence of dirt or manure on the limb, which could create areas of reduced minimum temperature. However, such different outcomes for the various considered parameters could be of interest in future studies to see whether an interaction between the three parameters (Tmax, Tmean, and Tmin) could be capable of predicting with even higher reliability whether a limb is subjected to localized inflammation due to growth plate lesions.

The capability of IRT in distinguishing between healthy limbs and diseased ones was not diminished by the lack of intervention on the animals, such as necessity to clean the limbs prior investigation. In other studies, cleaning of the limbs was suspected to be necessary because of the influence of dirt on the reliability of thermography by affecting the surface’s ability to radiate absorbed energy (emissivity) and to conduct heat (conductivity) [21]. However, in the present study, differences between the affected and healthy limbs could be detected in an on-farm setting without the need to restrain the animals that were free in their boxes, and without cleaning the examined limbs before IRT imaging, thus indicating IRT as a reliable and fast on-field method.

Currently, the recognized standard measure to assess lameness in cattle is locomotion scoring [31]. However, routinely locomotion scoring an entire herd is both lengthy and an inherently subjective process [21,31,32,33]. Moreover, this technique requires the regular observation of the animals, which is both logistically and economically challenging in practice. An automated IRT imaging system, placed in an area of the barn with a high daily influx of animals (e.g., near drinking troughs/feeders) and connected to a remote ear tag detection system, could represent an aiding tool for the detection of locomotory apparatus diseases in the herd. The development of a computerized system screening bovine limbs on a regular basis could represent an innovative approach for detecting lameness problems. This automatic detection system could signal animals that are developing pathologies of the musculoskeletal system, allowing for the rapid intervention for closer inspection and treatment.

However, one of the limits of IRT is in distinguishing between different lesion etiologies such as aseptic epiphysitis [6,7] and septic osteitis and osteomyelitis [8,14] and lesion severity. IRT was seen effective as a detection tool for unspecified lesions, but its potential as a diagnostic test has yet to be proven. The surface skin temperature is likely to vary with the stage and severity of disease, and with different types of disease, a subject that warrants further investigation. Future research following the development of lesion cases longitudinally may establish temperature thresholds for early treatment interventions, and IRT could be used also to quantify treatment effectiveness, following recovery and recurrence.

## 5. Conclusions

The presence of growth plate lesions was detected using IRT. Growth plate lesions caused an elevated mean and maximum temperature both on the growth plate area (AR01) and on the metatarsus in its totality (AR02), compared to the limbs with no lesions.

The present study established the potential of IRT as a reliable, practical tool for detecting growth plate lesions. The fact that this can be performed without the need to clean the limbs, no animal restraint, and no lengthy X-ray procedures would enable practical application on farms. This represents an interesting alternative for clinical evaluations of bovine limbs, as this method is non-invasive, requires no contact with the evaluated animal, and allows for the collection of data in external environments.

Currently, such a technique has not been tested to distinguish between different kinds of lesions nor severity, and therefore future research is needed. However, IRT could represent a novel approach in the detection of locomotory apparatus diseases in beef bulls. This technique could be developed to ease screening procedures and could also be used to quantify the effectiveness of early treatment. IRT is a promising tool for the management of heard health.

## Figures and Tables

**Table 1:** Descriptive statistics of all parameters considered for AR01 and AR02 of the animals in the study.

ROI	Parameters	Mean ± SD
AR01	T°min (°C)	24.43 ± 5.88
	T°max (°C)	31.50 ± 3.78
	ΔT°max − min (°C)	7.08 ± 3.25
	T°mean (°C)	28.79 ± 4.53
AR02	T°min (°C)	21.46 ± 7.25
	T°max (°C)	31.86 ± 3.77
	ΔT°max − min (°C)	10.40 ± 4.41
	T°mean (°C)	27.95 ± 5.30

**Table 2:** Mean ± standard deviation of AR01(growth plate area) and AR02 (whole metatarsus area) parameters among healthy and diseased limbs.

ROI	Parameters	Healthy Limb	Affected Limb	Difference between Affected and Healthy Measures (Least Sq Means (95% CI)
AR01	T°min (°C)	24.43 ± 5.88	25.31 ± 6.03 *	1.8 (0.58–2.95)
	T°max (°C)	31.50 ± 3.78	32.51 ± 3.21 **	2.0 (1.11–2.93)
	ΔT°max − min (°C)	7.08 ± 3.25	7.2 ± 3.39	0.3 (1.06–1.56)
	T°mean (°C)	28.79 ± 4.53	29.72 ± 4.36 **	1.9 (1.11–2.64)
AR02	T°min (°C)	21.46 ± 7.25	22.44 ± 7.08	1.9 (0.59–3.34)
	T°max (°C)	31.86 ± 3.77	32.62 ± 3.09 *	1.5 (0.52–2.50)
	ΔT°max − min (°C)	10.40 ± 4.41	10.17 ± 4.50	−0.5 (−1.80–0.86)
	T°mean (°C)	27.95 ± 5.30	28.82 ± 4.96 **	1.8 (0.98–2.52)

* = *p* < 0.005, ** = *p* < 0.0001.

## Data Availability

The datasets used and/or analyzed during the current study are available from the corresponding author on reasonable request.
